# Supplementary material for: The interactome of intact mitochondria by cross-linking mass spectrometry provides evidence for coexisting respiratory supercomplexes
Source: Mol Cell Proteomics. 2017 Dec 8;17(2):216–32. doi: 10.1074/mcp.RA117.000470 (PMC5795388; doi:10.1074/mcp.RA117.000470)
Supplement: Supplemental Data [file supp_RA117.000470_133922_0_supp_23978_fzffwf.zip › spectra_annotation/mito_DR_spectra_annotation/470-1-2-1-22-1.pdf]

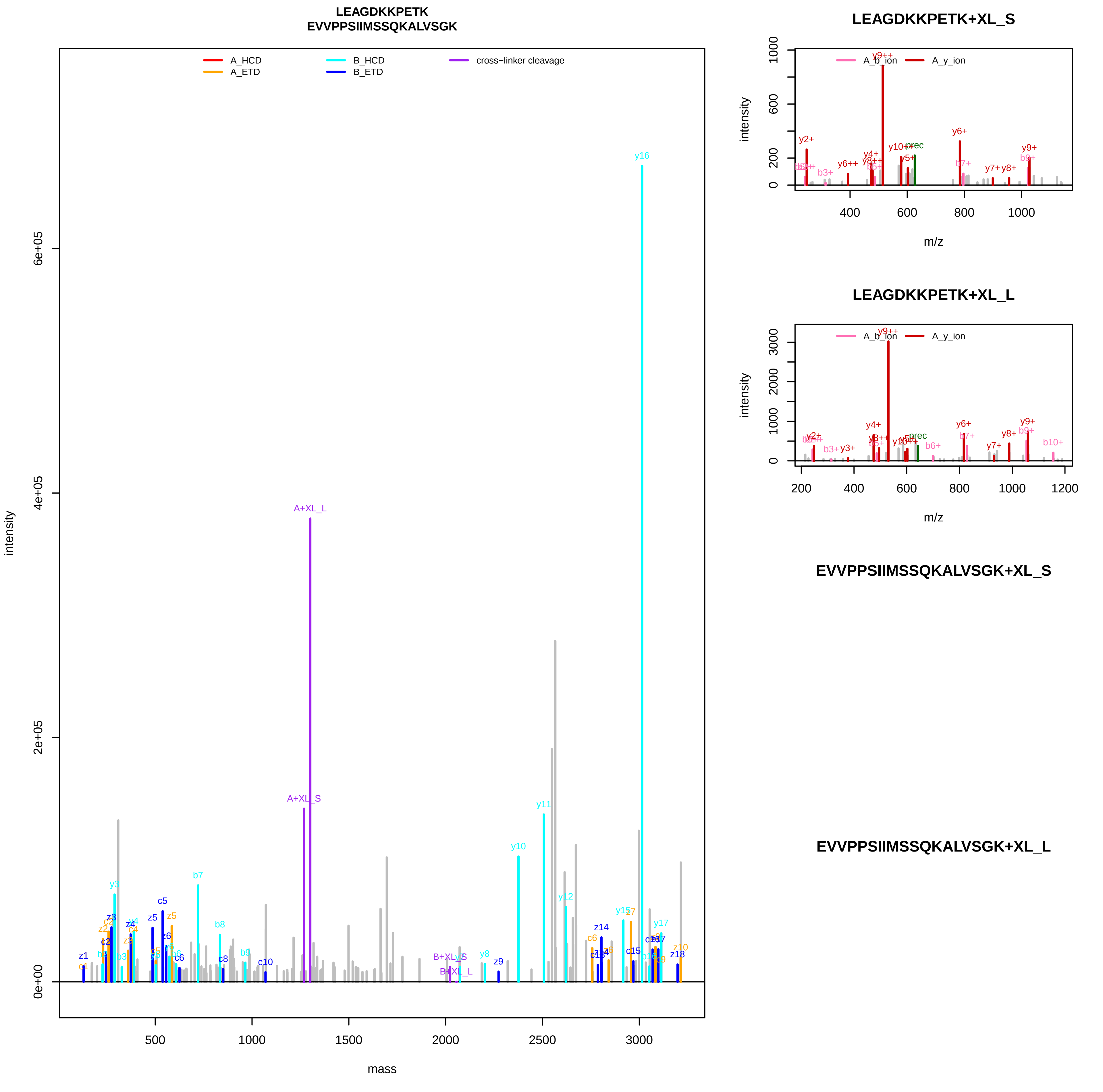
LEAGDKKPETK
EVVPPSIIMSSQKALVSGK
A_HCD
A_ETD
B_HCD
B_ETD
cross−linker cleavage
A+XL_L
A+XL_S
B+XL_S
B+XL_L
y16
y11
y10
y12
y15
y17
b7
b8
c5
z5
z3
z4
y3
z14
c15
z18
z10
c6
z9
y8
c10
c8
c6
0e+00
2e+05
4e+05
6e+05
intensity
500
1000
1500
2000
2500
3000
mass
LEAGDKKPETK+XL_S
A_b_ion
A_y_ion
y9++
y2+
y6+
y9+
y10++
y6++
b3+
prec
b7+
y7+
y8+
b9+
0
200
600
1000
400
600
800
1000
m/z
LEAGDKKPETK+XL_L
y4+
y3+
b6+
b10+
0
1000
2000
3000
200
400
600
800
1000
1200
EVVPPSIIMSSQKALVSGK+XL_S
EVVPPSIIMSSQKALVSGK+XL_L
